# Development and validation of a novel blending machine learning model for hospital mortality prediction in ICU patients with Sepsis

**DOI:** 10.1186/s13040-021-00276-5

**Published:** 2021-08-16

**Authors:** Zhixuan Zeng, Shuo Yao, Jianfei Zheng, Xun Gong

**Affiliations:** 1 Department of Emergency Medicine, Second Xiangya Hospital, Central South University, No.139, Middle Renmin Road, Changsha, 410011 Hunan Province China; 2 Emergency Medicine and Difficult Diseases Institute, Second Xiangya Hospital, Central South University, No.139, Middle Renmin Road, Changsha, 410011 Hunan Province China

**Keywords:** Sepsis, Intensive care unit, Hospital mortality prediction, Machine learning, MIMIC-III, eICU-CRD

## Abstract

**Background:**

Early prediction of hospital mortality is crucial for ICU patients with sepsis. This study aimed to develop a novel blending machine learning (ML) model for hospital mortality prediction in ICU patients with sepsis.

**Methods:**

Two ICU databases were employed: eICU Collaborative Research Database (eICU-CRD) and Medical Information Mart for Intensive Care III (MIMIC-III). All adult patients who fulfilled Sepsis-3 criteria were identified. Samples from eICU-CRD constituted training set and samples from MIMIC-III constituted test set. Stepwise logistic regression model was used for predictor selection. Blending ML model which integrated nine sorts of basic ML models was developed for hospital mortality prediction in ICU patients with sepsis. Model performance was evaluated by various measures related to discrimination or calibration.

**Results:**

Twelve thousand five hundred fifty-eight patients from eICU-CRD were included as the training set, and 12,095 patients from MIMIC-III were included as the test set. Both the training set and the test set showed a hospital mortality of 17.9%. Maximum and minimum lactate, maximum and minimum albumin, minimum PaO2/FiO2 and age were important predictors identified by both random forest and extreme gradient boosting algorithm. Blending ML models based on corresponding set of predictors presented better discrimination than SAPS II (AUROC, 0.806 vs. 0.771; AUPRC 0.515 vs. 0.429) and SOFA (AUROC, 0.742 vs. 0.706; AUPRC 0.428 vs. 0.381) on the test set. In addition, calibration curves showed that blending ML models had better calibration than SAPS II.

**Conclusions:**

The blending ML model is capable of integrating different sorts of basic ML models efficiently, and outperforms conventional severity scores in predicting hospital mortality among septic patients in ICU.

**Supplementary Information:**

The online version contains supplementary material available at 10.1186/s13040-021-00276-5.

## Background

Sepsis is a syndrome characterized by infection and infection-induced organ dysfunction. The Third International Consensus Definition for Sepsis (Sepsis-3) updated the definition as a “life-threatening organ dysfunction caused by a dysregulated host response to infection”, and recommended usage of the Sequential Organ Failure Assessment (SOFA) score instead of the Systemic Inflammatory Response Syndrome (SIRS) score for identifying organ dysfunction [1]. With an increasing incidence and prevalence over the last decades, sepsis has become the most common cause of admission to intensive care unit (ICU) and in-hospital death [2–5]. Despite considerable advances in diagnosis and management of sepsis, it is still a great clinical challenge due to a high mortality rate (about 20–30%), even in developed countries [3, 6]. And the care for sepsis results in an economic burden on individuals, families and health care systems [2].

Early prediction of hospital mortality is crucial for evaluating severity of illness and initiating adequate treatment for ICU patients with sepsis. Since it is difficult for clinicians to accurately predict hospital mortality of critically ill patients by an initial intuition, several severity scores have been developed for this purpose in the past decades, such as the Acute Physiology and Chronic Health Evaluation (APACHE), the Simplified Acute Physiology Score (SAPS) and the Mortality Probability Models (MPM) [7]. These conventional severity scores generally base on a multivariable logistic regression model, and predict hospital mortality using patient’s clinical characteristics measured within the first 24 h of the ICU stay. Despite wide application in clinic, these severity scores show limited performance since the logistic regression algorithm assumes that a linear and additive combination of predictors is reasonable for computing the outcome mathematically. However, that is not competent for simulating the real situation.

Machine learning (ML) is an interdiscipline of mathematics (probability theory, statistics) and computer science, and focuses on how computer learn from large data sets containing a multitude of variables. It is based on mathematical algorithms which identify potential patterns of data, and is implemented by computer programming which is capable of calculating a huge size of data. ML provides a novel approach for developing prediction model and has been gradually introduced into the medical field in recent years. One common application of ML is to predict mortality in various disease groups. In this study, we developed and validated a novel blending ML model, in which various ML algorithms were integrated, for hospital mortality prediction in ICU patients with sepsis.

## Methods

### Source of data

A retrospective cohort study was conducted in two large US-based ICU databases: the eICU Collaborative Research Database (eICU-CRD) [8] and the Medical Information Mart for Intensive Care III (MIMIC-III) [9]. The eICU-CRD is a multi-center ICU database comprising 200,859 ICU stays for 139,367 unique patients who were admitted to 335 ICUs in 208 hospitals located throughout the US during 2014 to 2015. And the MIMIC-III database is a single-center ICU database comprising 61,532 ICU stays for 46,476 unique patients who were admitted to 6 ICUs in the Beth Israel Deaconess Medical Center between 2001 and 2012. Both of the two databases provided comprehensive records of demographic characteristics, diagnoses, vital signs, laboratory tests, treatment information and other details from bedside monitors and nursing charts. These two databases were built with local ethical review board (ERB) approval and all tables of them were deidentified in accordance with the Health Insurance Portability and Accountability Act (HIPAA) standards, thus ERB approval from our institution was exempted. This study was conducted according to the Transparent Reporting of a multivariable prediction model for Individual Prognosis or Diagnosis (TRIPOD) [10].

### Participants

From both datasets, we included patients admitted to the ICU with sepsis, according to the sepsis-3 criteria [1]. Sepsis was defined as organ dysfunction consequent to an infection (or suspected infection). We firstly selected patients who met any of the following two criterion within a time window from 24 h before to 24 h after the ICU admission: 1. Having documented infection-related diagnoses according to the International Classification of Diseases, ninth revision, Clinical Modification (ICD-9-CM) codes provided by Angus et al. [2]; 2. Fulfilling the “suspected infection” criteria defined by Seymour CW et al. [11]. Then we identified consequent organ dysfunction as an acute change in SOFA ≥2 points within the first 24 h of ICU stay. In line with previous research [12–14], a baseline SOFA of zero was assumed for all patients since both these two datasets lack data prior to ICU admission. We included only the first ICU stay during a hospitalization and excluded patients with multiple hospitalizations to ensure a sample representing an independent patient for our prediction model. Besides, patients were excluded if any of the following exclusive criterion was met: 1. Age not between 16 and 89 years old; 2. ICU stay duration< 24 h; 3. Organ donor (liver, kidney, heart, cornea); 4. Missing rate of total variables (mentioned in the next section) more than 70%; 5. Missing survival status at discharge from hospital; 6. Obvious data error (e.g unrealistic extremum, wrong negative value). The outcome to be predicted in this study was the survival status at discharge from hospital. All eligible patients were included for development and validation of our ML model, without extra attempt to assess the appropriate sample size for this study.

### Predictors

A total of 65 variables, concerning demographic characteristics, vital signs, consciousness, laboratory tests, comorbidities, pivotal treatments and fluid balance within the first 24 h of an ICU stay, were extracted from these two databases as predictors. For some variables, both the maximum and minimum values within the first 24 h were collected considering that both abnormally high and low values could indicate severity of disease clinically. These 65 variables were referred to as the total variables in this study. And besides, three subsets of the total variables were selected in order to evaluate model performance. The first subset contained variables which were selected by a stepwise logistic regression model with both forward selection and backward elimination. The second subset contained variables applied in the SAPS II score. And the third subset contained variables applied in the SOFA score. The total variables and its three subsets were summarized in Table 1.

**Table 1:** Variables summary

Variable type	Variable
Demographic characteristics	age^a,b^, gender, ethnicity, BMI^a^, admission type^a,b^
Vital signs	max HR^b^, min HR^a,b^, max RR^a^, min RR^a^, max temperature^b^, min temperature^a^, max SBP^b^, min SBP^a,b^, max DBP, min DBP, max MAP, min MAP^a,c^, max SpO2, min SpO2^a^
Consciousness	min GCS^a,b,c^
Laboratory tests	max pH^a^, min pH^a^, max PaO2, min PaO2^a^, max PaCO2^a^, min PaCO2, max bicarbonate, min bicarbonate^b^, min PaO2/FiO2^a,b,c^, max WBC^a,b^, min WBC^a,b^, max hematocrit, min hematocrit, max platelet, min platelet^a,c^, max hemoglobin^a^, min hemoglobin, max bilirubin^a,b,c^, min bilirubin^a^, max albumin^a^, min albumin^a^, max creatinine^a,c^, min creatinine, max BUN^b^, min BUN^a^, max sodium^b^, min sodium^a,b^, max potassium^b^, min potassium^a,b^, max glucose, min glucose, max lactate^a^, min lactate^a^, max PT^a^, min PT
Comorbidities	AIDS^a,b^, hematological tumor^a,b^, metastatic cancer^a,b^
Pivotal treatments	Mechanical ventilation^a,b,c^ (including invasive and noninvasive ventilation), Renal replacement therapy^a^, Max dose of vasoactive drugs (dopamine^c^, dobutamine^c^, epinephrine^c^, norepinephrine^c^)
Fluid balance	24 h Urine output^a,b,c^

*Abbreviations*: *BMI* body mass index, *HR* heart rate, *RR* respiratory rate, *SBP* systolic blood pressure, *DBP* diastolic blood pressure, *MAP* mean arterial pressure, *GCS* Glasgow Coma Scale, *WBC* white blood cell count, *PT* prothrombin time

^a^First subset: variables selected by stepwise regression model

^b^Second subset: SAPS II related variables

^c^Third subset: SOFA related variables

### Training set and test set

Each eligible patients represented a sample for ML model. In this study, samples from the eICU-CRD constituted the training set for model development, and samples from the MIMIC-III constituted the test set for model validation. Such design pattern ensured that the training set and the test set were independent in order to obtain a convincing evaluation on model performance.

### Conventional severity scores

Two widely used conventional severity scores, the SAPS II and SOFA scores, were selected as benchmarks for model validation. Individual predicted probability of death for the SAPS II score was calculated according to the following formula by its authors [15]:

$$\log \left[\frac{pr\left[ death\right]}{1- pr\left[ death\right]}\right]=-7.7631+0.0737\ast SAPS\ II+0.9971\ast \log \left(1+ SAPS\ II\right)$$

However, the SOFA score could not be directly used to calculate the probability of death. In fact, the SOFA score was initially developed for assessment of multiple organ dysfunction rather than for mortality prediction, but many clinicians tended to use it to estimate the risk of death. Thus, we trained a SOFA score based logistic regression model by the training set to map the SOFA score to predicted probability of death, and we defined it as the refitted SOFA.

### Blending machine learning model

The blending ML model was composed of two layers of basic ML models. The first layer comprised various ML models and the second layer was a single ML model. In this study, a total of 9 ML models were applied in the first layer, including logistic regression (LR), linear discriminant analysis (LDA), classification and regression tree (CART), Naive Bayes model (NB), K-nearest neighbors (KNN), multi-layer perceptron (MLP), support vector machine (SVM), random forest (RF) and extreme gradient boosting (XGB). And logistic regression was applied in the second layer. A brief introduction of these basic ML models was provided in Supplement A. Before training the blending model, hyperparameters of basic ML models in the first layer were tuned through an internal five-fold cross validation (CV) on the training set. And a set of hyperparameters corresponding to the highest mean area under the receiver operating characteristic curve (AUROC) in the five-fold CV was searched by a grid search strategy.

The process of training the blending model was as follows: firstly, the training set was randomly and equally divided into training set 1 and training set 2, then the training set 1 was used to train the nine basic ML models in the first layer so that these trained basic ML models were capable of predicting probability of in-hospital death of a new sample with original variables (items in the total variables mentioned before); secondly, these nine trained models predicted original samples in the training set 2 to produce nine probabilities for each sample; thirdly, the original training set 2 was transformed to a new training set 2 by replacing original variables with the nine predicted probabilities which were referred to as new variables; finally, the new training set 2 was used to train the LR model in the second layer so that this trained LR model was capable of predicting a new sample with new variables. After these four steps the blending model was trained. Notably, division of the training set was responsible for avoiding information leakage during model training. In the first step above, the first-layer basic ML models were trained on the training set 1 through fitting their predicted probabilities to the true label (survival status). Thus, the second-layer LR model should trained on another training set rather than the training set 1, ensuring that it did not use the label-fitted predicted probabilities as its new variables to fit the label.

**Fig. 1:**
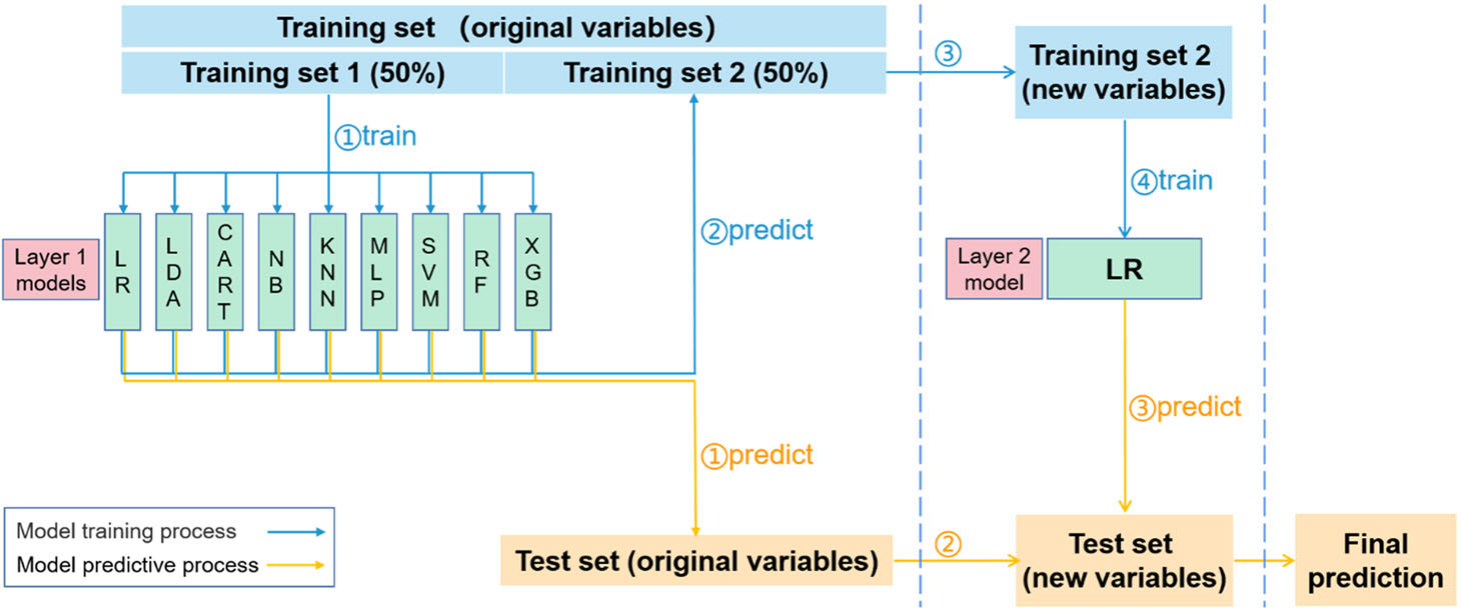
Blending machine learning model. Abbreviations: LR logistic regression, LDA linear discriminant analysis, CART classification and regression tree, NB Naive Bayes, KNN K-nearest neighbors, MLP multi-layer perceptron, SVM support vector machine, RF random forest, XGB extreme gradient boosting

Then the trained blending ML model was used to predict samples in the test set. Firstly, the trained basic models in the first layer predicted samples using their original variables; secondly, just like the third step of the model training process, the original test set was transformed to a new test set; finally, the trained LR model in the second layer predicted samples using their new variables and outputted the final predicted probabilities of in-hospital death. The processes of blending model training and prediction were summarized in Fig. 1.

### Data preprocess

KNN imputation [16] was applied to handle missing variables before model training and prediction. And besides, standard scale, which handled variables by removing the mean and scaling to unit variance, was applied in LR, LDA, NB, KNN, MLP, SVM. Standard scale was unnecessary for decision tree models such as CART, RF and XGB.

### Statistical analysis

Baseline characteristics between the training set and the test set were compared using either Student t test, rank-sum test or Chi-square test as appropriate. Continuous variables were described as mean (standard deviation) or median [interquartile range], and categorical variables were described as number (percentage).

Model performance was evaluated by various measures. AUROC was applied to assess discrimination of a model. DeLong’s test [17] was used to compare AUROCs between different models. Calibration curve, which was able to show the proximity between predicted mortality and actual mortality, was applied to assess model calibration. The more conventional Hosmer-Lemeshow statistic was abandoned considering its limited performance in large samples [18]. Area under the precision-recall curve (AUPRC) was also applied since it was valuable for unbalanced data set. The other measures included accuracy, recall, precision and F1 score. In order to estimate the influence of different variable sets to model performance, we built four blending ML models based on the total variables, the variables selected by stepwise logistic regression model, SAPS II related variables and SOFA related variables. And they were named as BM_total, BM_reg, BM_SAPSII and BM_SOFA respectively. To reduce randomness in single division of the training set, model training and prediction for all these blending ML models were repeated 10 times and mean and 95% confidence interval (CI) of these measurements were calculated. Besides, in order to compare predictive performance between blending ML model and conventional severity scores, the BM_SAPSII was compared to the SAPS II score and the BM_SOFA was compared to the refitted SOFA.

ML models were built using the scikit-learn package version 0.23.1, a machine learning package based on Python. Statistical analyses were performed using IBM SPSS Statistics software version 25.0. Two tailed *P* < 0.05 was considered as statistical significance.

## Results

### Participants

**Fig. 2:**
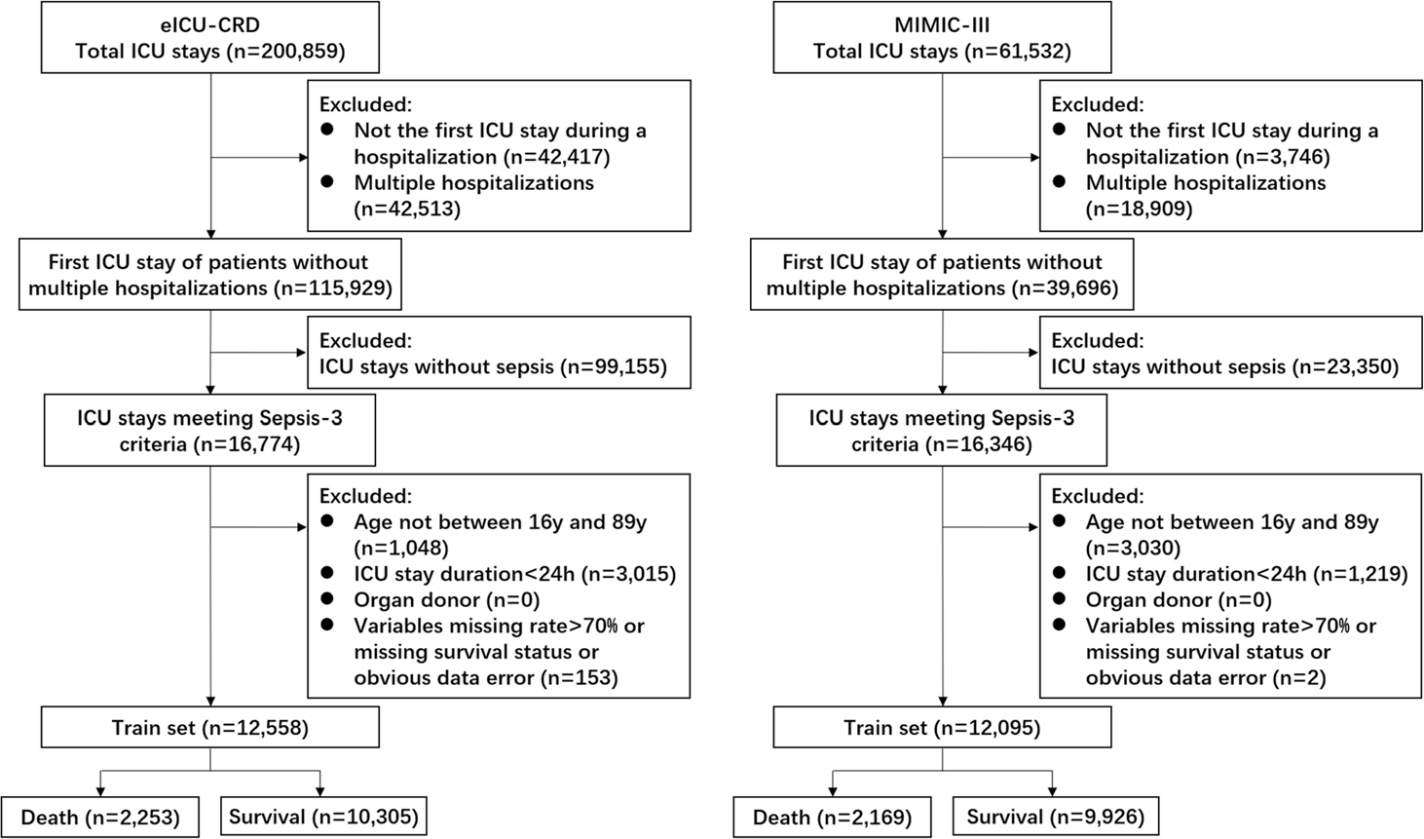
Flow chart of sample inclusion

Twelve thousand five hundred fifty-eight patients in the eICU-CRD were included as the training set, and 12,095 patients in the MIMIC-III were included as the test set (Fig. 2). Number of enrolled hospitals, distribution of ICU type, SOFA score, length of ICU stay and hospital mortality were presented in Table 2. There were significant statistical differences between the training set and the test set in SOFA score (5.3 vs. 5.5) and length of ICU stay (70.0 h vs. 74.0 h). As shown in Table 2, naming methods of ICU type were not identical between these two databases, thus ICU type was not selected as a predictor. Comparisons of the total variables were summarized in Supplement B. Notably, most of the total variables had significant statistical differences between the training set and the test set.

**Table 2:** Baseline characteristics between training set and test set

	Train set		Test set		*P*-value
Data source	eICU-CRD		MIMIC-III		
Number of hospitals	198		1		
Number of patients	12,558		12,095		
Unit type, n (%)	Med-Surg ICU	8255 (65.7)	MICU	4938 (40.8)	
	MICU	1479 (11.8)	SICU	1839 (15.2)	
	CCU	927 (7.4)	CCU	1328 (11.0)	
	CCU-CTICU	775 (6.2)	CSRU	2459 (20.3)	
	SICU	541 (4.3)	TSICU	1531 (12.7)	
	NICU	302 (2.4)			
	CSICU	135 (1.0)			
	CTICU	144 (1.1)			
SOFA score (mean (SD))	5.3 (2.8)		5.5 (3.1)		< 0.001
Length of ICU stay (hours, median [IQR])	70.0 [43.0, 127.0]		74.0 [43.9, 155.8]		< 0.001
Hospital mortality (%)	17.9		17.9		0.999

*Abbreviations*: *SD* standard deviation, *IQR* interquartile range, *CCU* cardiac care unit, *CSICU* cardiac surgical intensive care unit, *CSRU* cardiac surgery recovery unit, *CTICU* cardiothoracic intensive care unit, *MICU* medical intensive care unit, *NICU* neuro-intensive care unit, *SICU* surgical intensive care unit, *TSICU* trauma surgical intensive care unit

### Stepwise logistic regression model

The stepwise logistic regression model selected a total of 34 variables as predictors. As shown in Table 3, advanced age (OR for every 10 years increment, 1.269; 95% CI, 1.219 to 1.322), admission type of internal medicine (OR referring to unscheduled surgery, 1.526; 95% CI, 1.185 to 1.964), mechanical ventilation (OR, 1.687; 95% CI, 1.506 to 1.891), renal replacement therapy (OR, 1.730; 95% CI, 1.274 to 2.349), higher minimum lactate (OR, 1.120; 95% CI, 1.067 to 1.176), higher maximum lactate (OR, 1.072; 95% CI, 1.043 to 1.102) and comorbidities such as metastatic cancer (OR, 2.593; 95% CI, 1.858 to 3.618) were associated with increased probability of in-hospital death. Conversely, higher minimum albumin (OR, 0.848; 95% CI, 0.720 to 0.999), higher maximum albumin (OR, 0.679; 95% CI, 0.578 to 0.799), higher Glasgow Coma Scale (GCS) (OR, 0.943; 95% CI, 0.927 to 0.960) and increased urine output (OR for every 200 ml increment, 0.973; 95% CI, 0.963 to 0.983) were associated with decreased probability of in-hospital death.

**Table 3:** Variable selection of stepwise logistic regression model

Variables	OR [95% CI]	*P*-value
Demographic characteristics		
BMI	0.986 [0.979, 0.993]	< 0.001
Age (with each 10 years increment)	1.269 [1.219, 1.322]	< 0.001
Admission type (unscheduled surgery as reference)		
Internal medicine	1.526 [1.185, 1.964]	0.001
Scheduled surgery	0.936 [0.504, 1.738]	0.834
Comorbidities		
AIDS	2.281 [1.343, 3.873]	0.002
Hematological tumor	2.279 [1.661, 3.126]	< 0.001
Metastatic cancer	2.593 [1.858, 3.618]	< 0.001
Vital signs		
Minimum HR (with every 10 beats/min increment)	1.110 [1.073, 1.147]	< 0.001
Maximum RR	1.016 [1.009, 1.022]	< 0.001
Minimum RR	1.022 [1.012, 1.033]	< 0.001
Minimum temperature	0.811 [0.766, 0.858]	< 0.001
Minimum SBP (with every 10 mmHg increment)	0.922 [0.880, 0.966]	0.001
Minimum MAP (with every 10 mmHg increment)	0.927 [0.872,0.986]	0.016
Minimum SpO2	0.981 [0.976, 0.986]	< 0.001
Laboratory tests		
Minimum PaO2 (with every 10 mmHg increment)	1.020 [1.005, 1.035]	0.010
Maximum PaCO2 (with every 10 mmHg increment)	1.075 [1.041, 1.109]	< 0.001
Minimum PaO2/FiO2 (with every 20 mmHg increment)	0.940 [0.926, 0.955]	< 0.001
Maximum WBC	0.989 [0.979, 0.998]	0.023
Minimum WBC	1.021 [1.009, 1.034]	0.001
Minimum platelet (with every 20*10^12^/L increment)	0.981 [0.971, 0.992]	0.001
Maximum hemoglobin	0.942 [0.918, 0.966]	< 0.001
Minimum bilirubin	1.073 [1.048, 1.098]	< 0.001
Maximum albumin	0.679 [0.578, 0.799]	< 0.001
Minimum albumin	0.848 [0.720, 0.999]	0.049
Maximum creatinine (with every 0.1 mg/dl increment)	0.991 [0.987, 0.996]	< 0.001
Minimum BUN	1.009 [1.006, 1.012]	< 0.001
Minimum sodium (with every 10 mmol/L increment)	0.900 [0.830, 0.975]	0.010
Minimum potassium	1.110 [1.018, 1.211]	0.019
Maximum lactate	1.072 [1.043, 1.102]	< 0.001
Minimum lactate	1.120 [1.067, 1.176]	< 0.001
Maximum PT	1.013 [1.006, 1.020]	< 0.001
Minimum GCS	0.943 [0.927, 0.960]	< 0.001
Mechanical ventilation	1.687 [1.506, 1.891]	< 0.001
Renal replacement therapy	1.730 [1.274, 2.349]	0.037
24 h urine output (with every 200 ml increment)	0.973 [0.963, 0.983]	< 0.001

*Abbreviations*: *OR* odds ratio, *CI* confidence interval, *BMI* body mass index, *HR* heart rate, *RR* respiratory rate, *SBP* systolic blood pressure, *MAP* mean arterial pressure, *GCS* Glasgow Coma Scale, *WBC* white blood cell count, *PT* prothrombin time

### Model performance

**Fig. 3:**
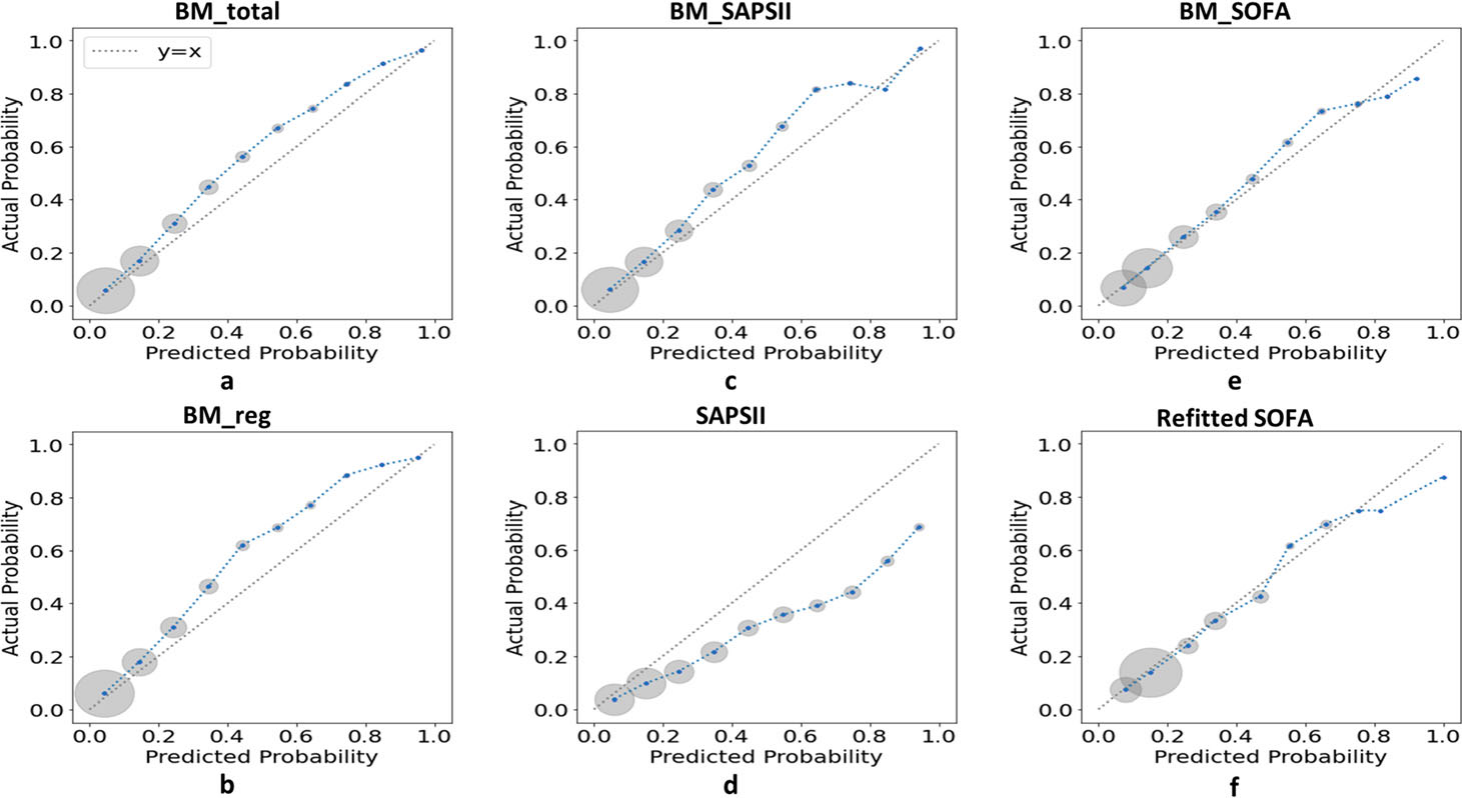
Calibration curves for external validation on the test set. For each model, the calibration curve was drew by dividing predicted probabilities into ten subgroups according to deciles of the [0,1] interval and plotting mean predicted probability versus mean actual probability for each subgroup. As shown, each blue point of a calibration curve represented a subgroup and the size of the gray circle around represented sample size of this subgroup. The dotted line was the identity line of y = x representing perfect calibration. The closer a calibration curve was to the identity line, the more similar predicted mortality was to actual mortality, indicating a better calibration of a model. Abbreviations: SAPS II Simplified Acute Physiology Score II, SOFA Sequential Organ Failure Assessment, BM_total blending model based on the total variables, BM_reg blending model based on variables selected by stepwise regression model, BM_SAPSII blending model based on SAPS II related variables, BM_SOFA blending model based on SOFA related variables

Predictive performance of different blending ML models, SAPS II and refitted SOFA was evaluated by an external validation (EV) on the test set. All the numerical measures were summarized in Table 4 and the graphical calibration curves were showed in Fig. 3. Comparisons of AUROCs between models were provided in the last column of Table 4. The BM_total and the BM_reg showed the best discrimination according to their AUROCs (0.815; 95% CI, 0.808 to 0.822 vs. 0.817; 95% CI, 0.810 to 0.823, *P* = 0.251). However, quantity of variables used in the BM_total was much more than that in the BM_reg (65 vs. 34). The BM_reg showed a significantly greater AUROC than the BM_SAPII and the BM_SOFA (0.817; 95% CI, 0.810 to 0.823 vs. 0.806; 95% CI, 0.799 to 0.813, *P* < 0.001 and 0.742; 95% CI, 0.734 to 0.749, *P* < 0.001, respectively). For comparing AUROCs between blending ML model and conventional severity score, the BM_SAPSII outperformed the SAPS II score (0.806; 95% CI, 0.799 to 0.813 vs. 0.771; 95% CI, 0.764 to 0.779, *P* < 0.001), and the BM_SOFA outperformed the refitted SOFA (0.742; 95% CI, 0.734 to 0.749 vs. 0.706; 95% CI, 0.698 to 0.714, P < 0.001). And in terms of comparing AUPRCs, the results were consistent with AUROCs. Figure 2 showed that the calibration curves of the blending ML models and the refitted SOFA presented a better fitness to the identity line than that of the SAPS II.

**Table 4:** Predictive performance of blending models, SAPS II and refitted SOFA on test set

	Accuracy [95% CI]	Recall [95% CI]	Precision [95% CI]	F1 score [95% CI]	AUPRC [95% CI]	AUROC [95% CI]	*P*-value for comparison between AUROCs
BM_total	0.845 [0.844, 0.846]	0.203 [0.190, 0.216]	0.748 [0.737, 0.758]	0.318 [0.302, 0.335]	0.536 [0.534, 0.539]	0.815 [0.808, 0.822]	vs. BM_reg: *p* = 0.251
BM_reg	0.845 [0.844, 0.845]	0.192 [0.183, 0.201]	0.768 [0.759, 0.776]	0.306 [0.295, 0.318]	0.542 [0.540, 0.544]	0.817 [0.810, 0.823]	vs. BM_SAPSII: *p* < 0.001 vs. BM_SOFA: *p* < 0.001
BM_SAPSII	0.842 [0.841, 0.844]	0.198 [0.179, 0.216]	0.726 [0.706, 0.746]	0.309 [0.286, 0.331]	0.515 [0.511, 0.518]	0.806 [0.799, 0.813]	vs. SAPSII: *p* < 0.001
BM_SOFA	0.835 [0.834, 0.836]	0.154 [0.145, 0.163]	0.679 [0.668, 0.690]	0.251 [0.239, 0.262]	0.428 [0.426, 0.431]	0.742 [0.734, 0.749]	vs. SOFA: *p* < 0.001
SAPS II	0.798	0.458	0.438	0.448	0.429	0.771 [0.764, 0.779]	
Refitted SOFA	0.833	0.130	0.680	0.218	0.381	0.706 [0.698, 0.714]	

*Abbreviations*: *AUPRC* area under the precision-recall curve, *AUROC* area under the receiver operating characteristic curve, *CI* confidence interval, *SAPS II* Simplified Acute Physiology Score II, *SOFA* Sequential Organ Failure Assessment, *BM_total* blending model based on the total variables, *BM_reg* blending model based on variables selected by stepwise regression model, *BM_SAPSII* blending model based on SAPS II related variables, *BM_SOFA* blending model based on SOFA related variables

### Basic machine learning models

**Fig. 4:**
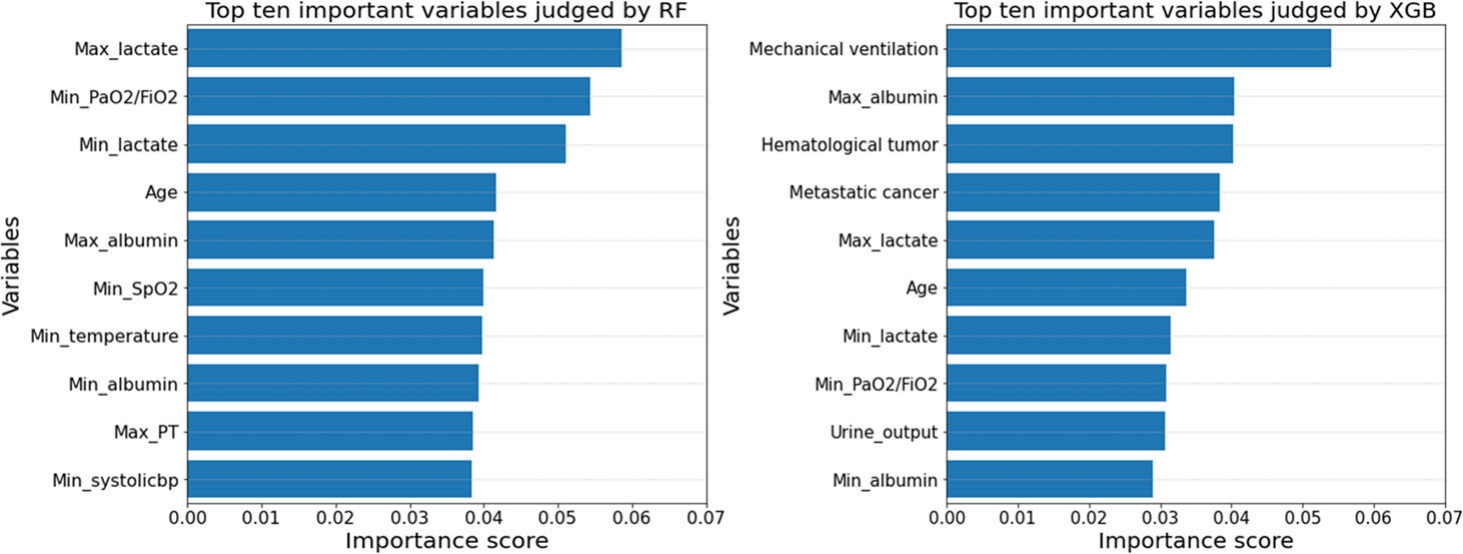
Variable importance derived from RF and XGB. Abbreviations: RF random forest, XGB extreme gradient boosting

For each basic ML model, major tuned hyperparameters were provided in Supplement C. Besides, Appendix C also demonstrated AUROCs of all the basic ML models in internal five-fold CV on the training set and AUROCs in EV on the test set. XGB showed the highest mean AUROC (0.805; 95% CI, 0.801 to 0.809) in internal five-fold CV and the highest AUROC (0.814; 95% CI, 0.807 to 0.821) in EV. And CART showed the lowest mean AUROC (0.733; 95% CI, 0.731 to 0.736) in five-fold CV and the lowest AUROC (0.711; 95% CI, 0.703 to 0.719) in EV. Among these basic ML algorithms, RF and XGB were capable of evaluating variable importance according to the sum of times that a variable was used to classify death versus survival in all basic trees of RF/XGB. Variable importance was assessed and the top 10 variables judged by both the two algorithms were showed in Fig. 4. Maximum and minimum lactate, maximum and minimum albumin, minimum PaO2/FiO2, and age were included into the most 10 important variables by both RF and XGB.

## Discussion

Hospital mortality prediction in ICU patients with sepsis has become an issue of great concern due to its guiding significance for clinicians. In this study, we developed and validated a novel blending ML model for this issue, and meanwhile we screened out various variables which were associated with hospital mortality of septic patients in ICU. Compared to conventional severity scores, our blending ML model showed better performance both in terms of discrimination and calibration. Lactate, albumin, minimum PaO2/FiO2 and age were identified by both RF and XGB algorithm as top important predictors.

We reviewed previous studies concerning applications of ML models for predicting mortality of patients with sepsis. In 2011, Ribas VJ et al. demonstrated that SVM showed a higher prediction accuracy and AUROC compared to Lasso and ridge regression and APACHE II score [19]. In 2016, Taylor RA et al. assessed RF, CART and LR model for predicting mortality of septic patients in emergency department and reported an increased AUROC for RF [20]. In 2019, also in emergency department, Perng JW et al. applied KNN, SVM, RF and SoftMax and reported an improved AUROC compared to quick SOFA and SIRS [21]. In 2020, Yao RQ et al. focused on mortality prediction in postoperative septic patients in MIMIC-III and showed that XGB outperformed LR and SOFA score in terms of discrimination and calibration [22], and Kong GL et al. reported that gradient boosting machine (GBM) showed a better performance compared to LR, Lasso regression, RF and SAPS II for mortality prediction in septic patients in MIMIC-III [23]. Compared with these previous studies, our study has two major advantages: firstly, two large ICU databases are applied as sources of the training set and the test set respectively, ensuring that model validation is really independent from model development; secondly, the blending ML model developed in this study is capable of integrating various sorts of ML models.

This study is a retrospective cohort study on two large ICU databases. The multi-center eICU-CRD has been selected as source of the training set in this study because it provides multi-source data to be mined by ML models. Our statistical analyses show a heterogeneity of ICU septic patients between the eICU-CRD and the MIMIC-III. Despite approximate hospital mortality, there are significant statistical differences in SOFA score, length of ICU stay and most of the total variables between the training set and the test set (Table 2 and Supplement B). It is interpretable since data of these two septic cohorts is from different hospitals, periods and distributions of ICU types. Although the test set presents a certain degree of difference from the training set, all the basic ML models employed in this study represent good generalization abilities since there is not obvious decrement of AUROC in EV on the test set compared to internal five-fold CV on the training set (Supplement C).

Our study indicates that the blending model outperforms conventional severity scores in predicting hospital mortality among septic patients in ICU. Furthermore, it provides a flexible method to integrate different sorts of ML models. Although various ML algorithms have been applied for mortality prediction, there is no conclusion about that which algorithm possesses the best performance. An objective fact is that a single sort of algorithm will not always suitable for identifying all the different potential patterns in big data. According to previous studies mentioned above, ensemble algorithms, such as RF, XGB and GBM, seem to possess more potential for such a complicated predictive task. In essence, the blending model is also an ensemble algorithm. The main difference is that the blending model is competent to integrate various sorts of ML models through its concise two-layer architecture, while RF, XGB and GBM are composed of homogenous basic models (generally CART). Thus, the blending model has advantages on mining data patterns using more heterogeneous ML algorithms. To our best knowledge, such a blending model has not been reported to be used for mortality prediction before.

The training and predictive processes of the blending model are efficient. Both the first-layer and the second-layer basic ML models are trained once without repeated training, using mutually exclusive two subsets of the training set. Besides, in the case of sample size in our study, equal division of the training set does not trend to affect performance of blending model due to reduction of sample size for training basic models. In fact, our results indicate that the BM_total performs at least as well as the most excellent basic model (XBG model based on the total variables) which is trained using the total training set, in terms of AUROC (Table 4 and Appendix C). The blending model is convenient to be extended to other medical predictive tasks by altering the combination of basic models and even the second-lay model according to different situations.

In this study we also explored significant variables for predicting hospital mortality among septic patients in ICU. The set of total variables includes the most common clinical characteristics obtained in ICU routine work for facilitating application of the predictive model. While the 34 variables selected by stepwise logistic regression model can build a more concise blending model with comparable predictive performance and reduced computing cost. Furthermore, through selecting variables by stepwise logistic regression model and assessing variable importance by RF and XGB algorithms, two important variables, which are rarely included in conventional severity scores, are identified in our study. One is lactate in the first 24 h of ICU stay. Our result suggests that both higher maximum and minimum lactate indicate an increased hospital mortality. Previous studies also show that hyperlactatemia is associated with high mortality among septic patients and other critically ill patients [24–26]. A widely held view is that elevated serum lactate is due to anaerobic glycolysis induced by tissue hypoxia and/or hypoperfusion which is common in sepsis, particularly in septic shock. And tissue hypoxia and/or hypoperfusion are considered to be a major cause of organ failure and mortality. However, the pathophysiology and clinical significance of hyperlactatemia in sepsis is still not fully understanded [27]. The other variable is albumin. Our research suggests that both lower maximum and minimum albumin indicate an increased hospital mortality. This result is also supported by several previous studies [28–30]. As the most common protein in the human body, serum albumin has important physiological functions such as maintaining plasma osmotic pressure, buffering function and binding capacity. Although hypoalbuminemia seems to predict a poor prognosis, infusion of albumin has not been confirmed to be an effective therapy to improve mortality in septic patients [31, 32]. Thus, further research is needed to clarify the role of these two variables in sepsis.

This study has several limitations. Firstly, data missing inevitably exists in such a retrospective cohort study using large public databases. Data missing can affect model performance, and severe data missing may also exclude some potential valuable variables for prediction. Thus, prospective cohorts are needed for further model validation and model updating. Secondly, our predictive model is capable of providing clinicians an early estimation about hospital mortality, but it has limited ability of give real-time prediction throughout ICU stay duration. A dynamic predictive model based on time series of clinical variables is planned in our future work. Finally, in this study the predicted outcome merely included hospital mortality. Long term mortality and other important clinical complications is also needed to be investigated.

## Conclusions

In conclusion, the blending ML model is capable of integrating different sorts of basic ML models efficiently, and outperforms conventional severity scores in predicting hospital mortality among septic patients in ICU. The blending ML model show its application value, since it provides clinicians an early prediction of hospital mortality and subsequently prompts adequate treatments for high-risk patient. Besides, some important variables ignored by conventional severity scores are proved to be valuable for such predictive issue. Further prospective research is expected to validate and improve the model.

## Supplementary Information

**Additional file 1.**

**Additional file 2.**

**Additional file 3.**

## Data Availability

Data of the eICU-CRD is available on website at https://eicu-crd.mit.edu/, and data of the MIMIC-III is available on website at https://mimic.physionet.org/. The extracted data and programming code for model development in this study are available from the corresponding author on reasonable request.

## Abbreviations

APACHE: Acute Physiology and Chronic Health Evaluation
AUPRC: Area under the precision-recall curve
AUROC: Area under the receiver operating characteristic curve
CART: Classification and regression tree
CV: Cross validation
eICU-CRD: eICU Collaborative Research Database
ERB: Ethical review board
EV: External validation
GCS: Glasgow Coma Scale
HIPAA: Health Insurance Portability and Accountability Act
ICD-9-CM: International Classification of Diseases, ninth revision, Clinical Modification
KNN: K-nearest neighbors
LDA: Linear discriminant analysis
LR: Logistic regression
MIMIC-III: Medical Information Mart for Intensive Care III
ML: Machine learning
MLP: Multi-layer perceptron
MPM: Mortality Probability Models
NB: Naive Bayes
RF: Random forest
SAPS: Simplified Acute Physiology Score
SIRS: Systemic Inflammatory Response Syndrome
SOFA: Sequential Organ Failure Assessment
SVM: Support vector machine
TRIPOD: Transparent Reporting of a multivariable prediction model for Individual Prognosis or Diagnosis
XGB: Extreme gradient boosting
