# Supplementary material for: Development and validation of a novel blending machine learning model for hospital mortality prediction in ICU patients with Sepsis
Source: BioData Min. 2021 Aug 16;14:40. doi: 10.1186/s13040-021-00276-5 (PMC8365981; doi:10.1186/s13040-021-00276-5)
Supplement: Supplementary file 1 — Additional file 1. [file 13040_2021_276_MOESM1_ESM.docx]

**Further Supplementary Information for: Development and Validation of a Novel Blending Machine Learning Model for Hospital Mortality Prediction in ICU Patients with Sepsis**

1. **A brief introduction to basic machine learning models in blending model**

Logistic regression (LR): LR is a classical binary classification model that identifies an optimal linear and additive combination of variables with the greatest probability of detecting the observed outcome [30].

Linear discriminant analysis (LDA): LDA is a data mining algorithm that can be used for classification by finding a projection hyperplane that minimizes the interclass variance and maximizes the distance between the projected means of the classes [31].

Classification and regression tree (CART): CART is a nonparametric model that identifies a multilevel bifurcated procedure to divide total samples into mutually exclusive and exhaustive subgroups. An unclassified sample is assigned to the same class as majority of samples in the subgroup where it is assigned by CART [32].

Naive Bayes model (NB): NB classifies a given sample described by its feature vector to the most likely class by maximum likelihood estimation method. Calculation of NB model is simple since it assumes that each input variable is conditionally independent given the class [33].

K-nearest neighbors (KNN): KNN is an instance-based algorithm based on the underlying idea that an unclassified sample should be assigned to the same class to which the majority of its K most similar samples belongs. The Euclidean distance between two samples in the feature space is commonly calculated as the measurement of similarity. K nearest neighbors of the query sample are the K samples with minimum Euclidean distance to it [34].

Multi-layer perceptron (MLP): MLP is the simplest form of artificial neural networks, named from mimicking the way by which biological neural network operates. MLP is composed of many interconnected neurons that constitute a multi-layer architecture containing an input layer, several hidden layers and an output layer. A neuron represents a computing unit which calculates a weighted sum of input variables from its previous layer and outputs a value to its next layer through a specified activation function. A prediction is calculated out through a successive computational process of obtaining feature variables through an input layer, processing variables through several hidden layers and putting out a prediction through an output layer [35].

Support vector machine (SVM): SVM is characterized by finding a hyperplane to sort samples into two categories. The hyperplane in feature space is located as far as possible from the nearest sample of both categories. The introduction of kernel function enables SVM to distinguish the samples which are linearly inseparable in the original feature space, because it maps the original samples to a higher dimensional space, in which the hyperplane exists [36].

Random forest (RF): RF is a bagging ensemble algorithm. It is composed of many basic decision trees, which are trained using random subsets sampled from training set by a bootstrap method. And random selection of feature variables is also used when growing a decision tree. By injecting randomness into model training in such a pattern, RF has excellent ability to prevent over-fitting. All decision trees are trained in parallel, and a final prediction is derived from integrating these weak decision trees [37].

Extreme gradient boosting (XGB): XGB is an improved boosting ensemble algorithm composed of a series of basic classifiers (decision trees in this study). Unlike RF, XGB combines basic decision trees in series instead of in parallel, which means that decision trees are added iteratively to refit the residual of the previous model. A newly added decision tree in a round of iteration focuses more on the samples misclassified in previous iteration. XGB adds a regularization term to simplify the model, which makes it superior to original gradient boosting algorithm in preventing over-fitting [38].

1. **Total variables between train set and test set**

|  | Train set (n=12558) | Test set (n=12095) | P-value |
| --- | --- | --- | --- |
| **Demographic data** |  |  |  |
| Age (y, mean (SD)) | 65.7 (15.3) | 64.4 (16.3) | <0.001 |
| Gender (male), n (%) | 6593 (52.5) | 6988 (57.8) | <0.001 |
| BMI (kg/m^2^, mean (SD)) | 29.5 (9.3) | 28.8 (7.0) | <0.001 |
| **Ethnicity** |  |  |  |
| White, n (%)  Black, n (%)  Hispanic, n (%)  Asian, n (%)  Native, n (%)  Other/unknown, n (%) | 9753 (77.7)  1280 (10.2)  552 (4.4)  180 (1.4)  86 (0.7)  707 (5.6) | 8664 (71.6)  834 (6.9)  363 (3.0)  307 (2.5)  6 (0.0)  1921 (15.9) | <0.001 |
| **Admission type** |  |  |  |
| Medical, n (%)  UnscheduledSurgical, n (%)  ScheduledSurgical, n (%) | 11655 (92.8)  761 (6.1)  142 (1.1) | 8349 (69.0)  2137 (17.7)  1609 (13.3) | <0.001 |
| **Vital Signs** |  |  |  |
| Max HR (bpm, mean (SD)) | 110.8 (22.4) | 107.0 (21.0) | <0.001 |
| Min HR (bpm, mean (SD)) | 76.9 (16.8) | 72.8 (15.6) | <0.001 |
| Max RR (bpm, mean (SD)) | 30.1 (8.5) | 28.1 (6.7) | <0.001 |
| Min RR (bpm, mean (SD)) | 14.4 (5.4) | 12.5 (3.9) | <0.001 |
| Max temperature (℃, mean (SD)) | 37.5 (0.9) | 37.7 (0.9) | <0.001 |
| Min temperature (℃, mean (SD)) | 36.3 (0.9) | 36.1 (0.9) | <0.001 |
| Max SBP (mmHg, mean (SD)) | 143.2 (25.4) | 149.9 (24.2) | <0.001 |
| Min SBP (mmHg, mean (SD)) | 89.8 (19.4) | 89.0 (16.9) | 0.003 |
| Max DBP (mmHg, mean (SD)) | 86.8 (20.7) | 83.7 (18.1) | <0.001 |
| Min DBP (mmHg, mean (SD)) | 46.5 (12.8) | 42.9 (11.1) | <0.001 |
| Max MAP (mmHg, mean (SD)) | 101.2 (21.1) | 106.2 (28.8) | <0.001 |
| Min MAP (mmHg, mean (SD)) | 59.6 (14.3) | 56.6 (13.3) | <0.001 |
| Max SpO2 (%, median [IQR]) | 100 [99, 100] | 100 [100, 100] | <0.001 |
| Min SpO2 (%, median [IQR]) | 92 [88, 95] | 93 [90, 95] | <0.001 |
| Min GCS (median [IQR]) | 15 [12, 15] | 15 [13, 15] | <0.001 |
| **Laboratory Tests** |  |  |  |
| Max pH (mean (SD)) | 7.39 (0.09) | 7.43 (0.07) | <0.001 |
| Min pH (mean (SD)) | 7.31 (0.13) | 7.31 (0.11) | 0.040 |
| Max PaO2 (mmHg, median [IQR]) | 111.0 [80.0, 182.0] | 244.0 [134.0, 399.0] | <0.001 |
| Min PaO2 (mmHg, median [IQR]) | 72.8 [59.0, 94.0] | 87.0 [70.0, 116.0] | <0.001 |
| Max PaCO2 (mmHg, mean (SD)) | 48.9 (20.5) | 47.7 (13.8) | <0.001 |
| Min PaCO2 (mmHg, mean (SD)) | 38.8 (13.2) | 35.2 (8.3) | <0.001 |
| Max bicarbonate (mmol/L, mean (SD)) | 24.7 (6.6) | 24.6 (4.3) | 0.150 |
| Min bicarbonate (mmol/L, mean (SD)) | 22.0 (6.7) | 21.7 (4.9) | <0.001 |
| Min PaO2/FiO2 (mmHg, median [IQR]) | 166.7 [102.0, 250.0] | 182.0 [121.7, 263.5] | <0.001 |
| Max WBC (10^9^/L, median [IQR]) | 14.5 [10.0, 20.5] | 13.6 [10.0, 18.4] | <0.001 |
| Min WBC (10^9^/L, median [IQR]) | 10.7 [7.2, 15.2] | 9.9 [7.0, 13.4] | <0.001 |
| Max hematocrit (%, mean (SD)) | 36.4 (7.0) | 36.1 (5.9) | <0.001 |
| Min hematocrit (%, mean (SD)) | 31.9 (6.9) | 29.1 (6.2) | <0.001 |
| Max platelet (10^12^/L, median [IQR]) | 213 [151, 291] | 213 [156, 289] | 0.065 |
| Min platelet (10^12^/L, median [IQR]) | 175 [122, 244] | 170 [117, 238] | <0.001 |
| Max hemoglobin (g/dL, mean (SD)) | 12.0 (2.4) | 12.0 (2.0) | 0.003 |
| Min hemoglobin (g/dL, mean (SD)) | 10.4 (2.3) | 9.9 (2.1) | <0.001 |
| Max bilirubin (mg/dL, median [IQR]) | 0.7 [0.5, 1.2] | 0.8 [0.5, 1.8] | <0.001 |
| Min bilirubin (mg/dL, median [IQR]) | 0.6 [0.4, 1.0] | 0.7 [0.4, 1.5] | <0.001 |
| Max albumin (g/dL, mean (SD)) | 3.0 (0.7) | 3.1 (0.7) | <0.001 |
| Min albumin (g/dL, mean (SD)) | 2.7 (0.7) | 3.0 (0.7) | <0.001 |
| Max creatinine (mg/dL, median [IQR]) | 1.4 [0.9, 2.4] | 1.1 [0.8, 1.7] | <0.001 |
| Min creatinine (mg/dL, median [IQR]) | 1.1 [0.8, 1.8] | 0.9 [0.7, 1.3] | <0.001 |
| Max BUN (mg/dL, median [IQR]) | 29.0 [18.0, 46.0] | 22.0 [15.0, 36.0] | <0.001 |
| Min BUN (mg/dL, median [IQR]) | 23.0 [14.0, 38.0] | 18.0 [12.0, 30.0] | <0.001 |
| Max sodium (mmol/L, mean (SD)) | 139.5 (5.8) | 140.4 (4.9) | <0.001 |
| Min sodium (mmol/L, mean (SD)) | 135.9 (5.9) | 136.2 (5.2) | <0.001 |
| Max potassium (mmol/L, mean (SD)) | 4.5 (0.8) | 4.8 (1.0) | <0.001 |
| Min potassium (mmol/L, mean (SD)) | 3.8 (0.6) | 3.7 (0.6) | <0.001 |
| Max glucose (mg/dL, median [IQR]) | 156.0 [124.0, 209.0] | 163.0 [132.0, 206.0] | <0.001 |
| Min glucose (mg/dL, median [IQR]) | 113.0 [93.0, 140.0] | 104.0 [89.0, 126.0] | <0.001 |
| Max lactate (mmol/L, median [IQR]) | 2.2 [1.4, 3.8] | 2.4 [1.6, 3.8] | <0.001 |
| Min lactate (mmol/L, median [IQR]) | 1.5 [1.0, 2.2] | 1.4 [1.0, 2.0] | 0.637 |
| Max PT (sec, median [IQR]) | 15.4 [13.3, 19.3] | 14.9 [13.5, 17.3] | <0.001 |
| Min PT (sec, median [IQR]) | 14.9 [13.0, 17.7] | 13.8 [12.9, 15.2] | <0.001 |
| **Pivotal treatments** |  |  |  |
| Mechanical ventilation, n (%) | 5370 (42.8) | 7324 (60.6) | <0.001 |
| Renal replacement therapy, n (%) | 412 (3.3) | 471 (3.9) | 0.011 |
| Vasoactive drugs^*^ |  |  |  |
| dopamine, n (%) | 138 (1.1) | 778 (6.4) | <0.001 |
| dobutamine, n (%) | 82 (0.7) | 167 (1.4) | <0.001 |
| epinephrine, n (%) | 72 (0.6) | 495 (4.1) | <0.001 |
| norepinephrine, n (%) | 1538 (12.2) | 2102 (17.4) | <0.001 |
| **Comorbidities** |  |  |  |
| AIDS, n (%) | 93 (0.7) | 135 (1.1) | 0.003 |
| Hematological tumor, n (%) | 236 (1.9) | 431 (3.6) | <0.001 |
| Metastatic tumor , n (%) | 192 (1.5) | 431 (3.6) | <0.001 |
| Urine output (ml, median [IQR]) | 1250 [910, 1695] | 1669 [1000, 2535] | <0.001 |

Abbreviations: SD standard deviation, IQR interquartile range, BMI body mass index, HR heart rate, RR respiratory rate, SBP systolic blood pressure, DBP diastolic blood pressure, MAP mean arterial pressure, GCS Glasgow Coma Scale, WBC white blood cell count, PT prothrombin time

* Maximum dose of vasoactive drug was not presented since data is too sparse for statistical analysis. Instead, usage of vasoactive drug was presented as categorical variable in this table. But maximum dose of vasoactive drug was still used for model training.

1. **Optimized hyperparameters and AUROCs of basic models in internal five-fold CV and EV**

|  | Major optimized hyperparameters | 5-fold CV on train set  Mean AUROC [95%CI] | EV on test set  AUROC [95%CI] |
| --- | --- | --- | --- |
| LR | penalty='l1'; solver='liblinear'; C=1.0; class_weight='balanced' | 0.789 [0.786, 0.792] | 0.789 [0.781, 0.796] |
| LDA | none | 0.788 [0.785, 0.790] | 0.787 [0.780, 0.794] |
| CART | criterion='gini'; max_depth=7; min_samples_split=8; class_weight='balanced' | 0.733 [0.731, 0.736] | 0.711 [0.703, 0.719] |
| NB | none | 0.743 [0.736, 0.750] | 0.749 [0.741, 0.756] |
| KNN | n_neighbors=50; metric='minkowski'; weights='distance' | 0.754 [0.749, 0.760] | 0.753 [0.745, 0.761] |
| MLP | hidden_layer_sizes=(3, 3, 4); activation='relu'; solver='adam'; learning_rate_init=0.001 | 0.776 [0.771, 0.782] | 0.787 [0.780, 0.794] |
| SVM | kernel='rbf'; C=0.8; gamma=0.005 | 0.776 [0.773, 0.780] | 0.776 [0.769, 0.784] |
| RF | n_estimators=800; max_depth=14; min_samples_split=4; min_samples_leaf=2; max_features='sqrt'; | 0.790 [0.785, 0.795] | 0.798 [0.791, 0.805] |
| XGB | n_estimators=800; learning_rate=0.01; colsample_bytree=0.8; subsample=0.8; max_depth=11; min_child_weight=1; gamma=0.2 | 0.805 [0.801, 0.809] | 0.814 [0.807, 0.821] |

Abbreviations: LR logistic regression, LDA linear discriminant analysis, CART classification and regression tree, NB Naive Bayes, KNN K-nearest neighbors, MLP multi-layer perceptron, SVM support vector machine, RF random forest, XGB extreme gradient boosting, CV cross validation, EV external validation

**References**

1. Stoltzfus JC. Logistic regression: a brief primer. Acad Emerg Med. 2011;18(10):1099-104. doi:10.1111/j.1553-2712.2011.01185.x
2. Turnip A. Linear Discriminant Analysis. Robust Data Mining. Springer New York, 2013.
3. Lemon SC, Roy J, Clark MA, Friedmann PD, Rakowski W. Classification and regression tree analysis in public health: methodological review and comparison with logistic regression. Ann Behav Med. 2003;26(3):172-81. doi: 10.1207/S15324796ABM2603_02
4. Rish I. An empirical study of the naive Bayes classifier. Journal of Universal Computer Science. 2001;1(2):127.
5. Zhang Z. Introduction to machine learning: k-nearest neighbors. Ann Transl Med. 2016;4(11):218. doi: 10.21037/atm.2016.03.37
6. Hornik K, Stinchcombe M, White H. Multilayer feedforward networks are universal approximators. Neural Networks. 1989;2(5):359-66.
7. Noble WS. What is a support vector machine. Nat biotechnol. 2006;24(12):1565-67.
8. Breiman L. Random forests. Machine Learning. 2001;45:5-32.
9. Chen T, Guestrin C. Xgboost: A scalable tree boosting system. arXiv. New York: ACM Press; 2016.p.785-94.
